# The Biological Effects of Forsythia Leaves Containing the Cyclic AMP Phosphodiesterase 4 Inhibitor Phillyrin

**DOI:** 10.3390/molecules26082362

**Published:** 2021-04-19

**Authors:** Sansei Nishibe, Kumiko Mitsui-Saitoh, Junichi Sakai, Takahiko Fujikawa

**Affiliations:** 1 Faculty of Pharmaceutical Sciences, Health Sciences University of Hokkaido, Ishikari-Tobetsu, Hokkaido 061-0293, Japan; 2 Faculty of Health and Sport, Nagoya Gakuin University, 1350 Kamishinano, Seto, Aichi 480-1298, Japan; mitsuik@ngu.ac.jp (K.M.-S.); j_sakai@ngu.ac.jp (J.S.); 3 Faculty of Pharmaceutical Sciences, Suzuka University of Medical Science, 3500-3 Minamitamagaki-cho, Suzuka-City, Mie 513-8670, Japan

**Keywords:** forsythia leaves, polyphenolic compound, forsythiaside, phillyrin, PDE4 inhibitor, anti-obesity, atopic dermatitis, influenza A virus infection, phytoestrogen

## Abstract

Forsythia fruit (*Forsythia suspensa* Vahl (Oleaceae)) is a common component of Kampo medicines for treating the common cold, influenza, and allergies. The main polyphenolic compounds in the leaves of *F. suspensa* are pinoresinol β-d-glucoside, phillyrin and forsythiaside, and their levels are higher in the leaves of the plant than in the fruit. It is known that polyphenolic compounds stimulate lipid catabolism in the liver and suppress dyslipidemia, thereby attenuating diet-induced obesity and polyphenolic anti-oxidants might attenuate obesity in animals consuming high-fat diets. Recently, phillyrin was reported as a novel cyclic AMP phosphodiesterase 4 (PDE4) inhibitor derived from forsythia fruit. It was expected that the leaves of *F. suspensa* might display anti-obesity effects and serve as a health food material. In this review, we summarized our studies on the biological effects of forsythia leaves containing phillyrin and other polyphenolic compounds, particularly against obesity, atopic dermatitis, and influenza A virus infection, and its potential as a phytoestrogen.

## 1. Introduction

Obesity has become an urgent worldwide public health problem in recent decades. Diet-induced obesity is closely associated with lifestyle-related diseases (e.g., diabetes, hyperlipidemia, hypertension), which are primary risk factors for cardiovascular disease [1]. Therefore, preventing obesity is strongly encouraged to alleviate various lifestyle-related diseases.

*Forsythia suspensa* Vahl (Oleaceae) is listed in Japanese Pharmacopoeia as the original plant of the crude drug “forsythia fruit” [2], a common component in Kampo medicines for treating the common cold, influenza, and allergies. With at least 3000 years of continuous use, forsythia fruit is traditionally considered a detoxicant for treating so-called toxic and hot conditions. In addition, forsythia fruit is one of the main components of “Bofutsushosan” (BOFU), an extremely popular Kampo medicine in Japan with anti-obesity effects [3]. Wang et al. reported review with the title of “Phytochemistry, pharmacology, quality control, and future research of *Forsythia suspensa* (Thunb.) Vahl: A review” [4]. However, we could not find a description of the anti-obesity effects of the forsythia fruit or leaves alone in the review.

We found that the main polyphenolic compounds in leaves of *F. suspensa* are pinoresinol β-d-glucoside, phillyrin and forsythiaside and their levels are higher in the leaves of the plant than in its fruit [5,6]. Polyphenolic compounds stimulate lipid catabolism in the liver and suppress dyslipidemia, thereby attenuating diet-induced obesity, and polyphenolic anti-oxidants can attenuate obesity in animals fed a high-fat diets (HFD) [7,8]. We found that pinoresinol β-d-glucoside is a strong cyclic AMP phosphodiesterase (PDE) inhibitor in vitro [9]. However, it was unclear whether pinoresinol β-d-glucoside is a selective PDE4 inhibitor or not. Phillyrin also showed a moderate inhibition in our analysis. Phillyrin was recently reported as a novel selective PDE4 inhibitor [10]. As PDE4 inhibition is a therapeutic strategy for metabolic disorders [11], it is expected that the fruit and leaves of *F. suspensa* might display anti-obesity effects.

However, the use of forsythia fruit as a health food is currently prohibited in Japan by the Regulatory law “Distinction between drugs and food stuffs” from Ministry of Health, Labor, and Welfare (Japan). Therefore, we studied the biological effects of forsythia leaves for use as a health food material as an alternative to pharmaceuticals. In addition, studies of *F. suspensa* regarding its effects on atopic dermatitis [12] and the prevention of influenza [13,14] have been reported.

This review summarizes our studies on the biological effects of forsythia leaves containing phillyrin and other polyphenolic compounds against obesity, atopic dermatitis, and influenza A virus infection, as well as its potential as a phytoestrogen.

## 2. Structures of Phillyrin and Forsythiaside

Pinoresinol β-d-glucoside, phillyrin and forsythiaside (caffeoyl glycoside of 3,4-dihydroxy-β-phenethyl alcohol) were isolated as the main polyphenolic compounds from the fruit and leaves of *F. suspensa*, respectively [5,6]. The contents of the compounds in the leaves are as follows; pinoresinol β-d-glucoside, 0.3–2%, phillyrin, 0.4–3% and forsythiaside, 0.4–5% [6].

### 2.1. Phillyrin

**Figure 1:**
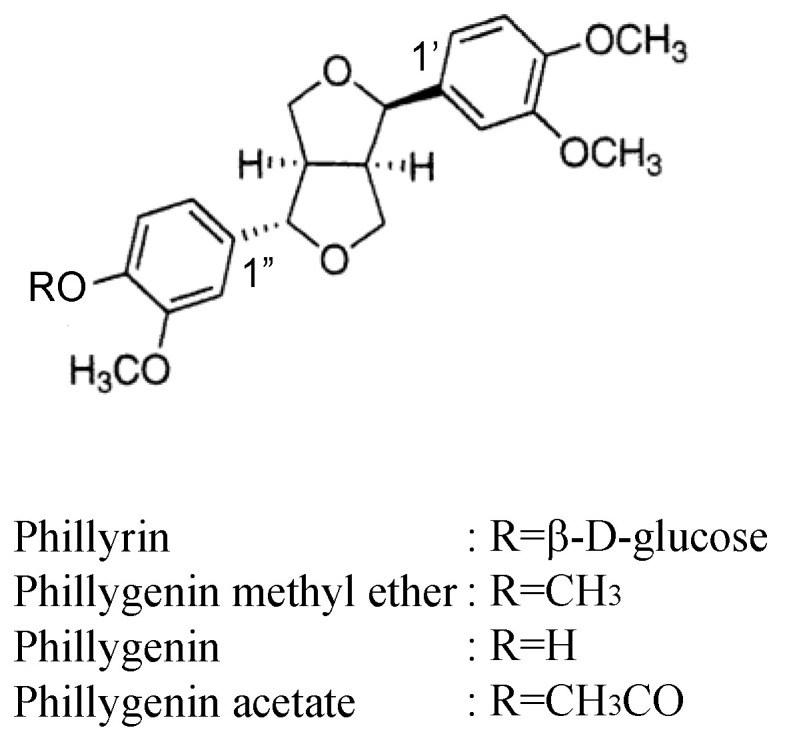
Chemical structures of phillyrin and aglycone derivatives.

Phillyrin, the glucoside of (+)-epipinoresinol monomethyl ether, is widely distributed in the leaves of *Forsythia* species [15]. The position (C-4′ or C-4″) of the glucose linkage with the aglycone was previously undetermined. We established the position using the ^13^C-NMR spectra of aglycone derivatives as shown in Figure 1 [16]. The aglycone was obtained via the hydrolysis of phillyrin by β-glucosidase. Pelter et al. reported that the 1′ and 1″ carbon atoms of the axial and equatorial aryl groups of 2,6-diaryl-3,7-dioxabicyclo [3.3.0] octane lignans are clearly distinct from each other in the ^13^C-NMR spectra, that is, the signals of former are assigned to approximately 131 ppm, whereas the latter signals are assigned to approximately 134 ppm for the veratryl group [17].

Comparing the spectral data of these three derivatives, appreciable differences of chemical shift values for C-1″ (equatorial aryl group) at 133.8 ppm for phillygenin methyl ether, 133.1 ppm for phillygenin and 140.3 ppm for phillygenin acetate were observed via aryl carbon shielding, because of the effect of the substituent at the *para* position, whereas differences for C-1′ (axial aryl group) at approximately 131 ppm were not observed. These results indicated that the glucose linkage in phillyrin is at the C-4″ position as shown in Table 1.

### 2.2. Forsythiaside

We isolated a novel caffeoyl glycoside of 3,4-dihydroxy-β-phenethyl alcohol for the first time and named the compound forsythiaside [18]. The ^1^H-NMR spectrum of acetate of forsythiaside revealed the presence of five alcoholic acetoxyl and four phenolic acetoxyl groups. Alkaline treatment of forsythiaside followed by acid hydrolysis gave caffeic acid and 3,4-dihydroxy-β-phenethyl alcohol, which were identified via comparison with authentic samples by gas chromatography (GC) and thin-layer chromatography (TLC). d-glucose and l-rhamnose were detected in the hydrolysate at a 1:1 ratio by GC. These data suggest that forsythiaside bears a marked structural resemblance to the acteoside (3,4-dihydroxy-β-phenethyl-*O*-α-l-rhamnopyranosyl(1→3)-4-*O*-caffeoyl-β-d-glucopyranoside), isolated from *Syringa vulgaris* (Oleaceae).

**Figure 2:**
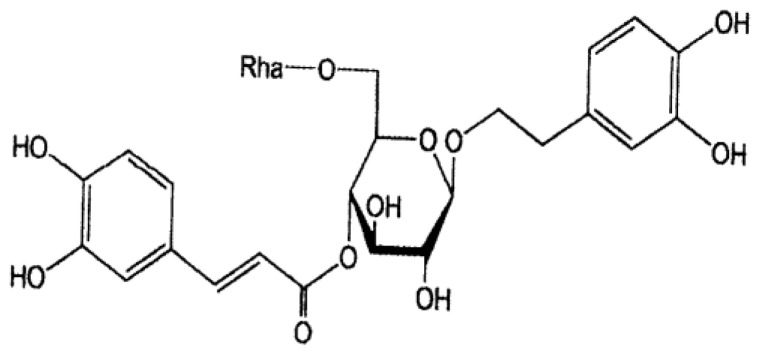
Chemical structure of forsythiaside.

The ^13^C-NMR spectrum of forsythiaside was compared with that of known compounds, i.e., 3,4-dihydroxy-β-phenethyl alcohol, rutin bearing a rutinose moiety and chlorogenic acid bearing a caffeate moiety. Based on the ^13^C-NMR data, namely the chemical shifts of the rutinose moiety of forsythiaside relative to that of rutin, the glycosidation shift of the α-carbon of forsythiaside relative to that of 3,4-dihydroxy-β-phenethyl alcohol, and the calculated chemical shift of the α-carbon of forsythiaside relative to that of 3,4-dihydroxy-β-phenethyl alcohol, the structure of forsythiaside was established as 3,4-dihydroxy-β-phenethyl-*O*-α-l-rhamnopyranosyl-(1→6)-4-*O*-caffeoyl-β-d-glucopyranoside as shown in Figure 2.

## 3. The Anti-Obesity Effects of Forsythia Leaves

**Figure 3:**
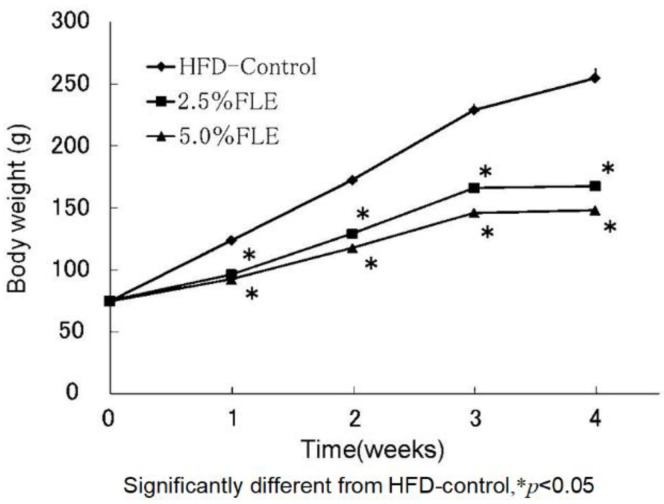
Effects of FLE on the body weight of rats.

Forsythia leaf water extract (FLE) was prepared from the leaves of *F. suspensa.* The contents of the main compounds in FLE were determined using HPLC as follows: pinoresinol β-d-glucoside 1.74 g/100 g, phillyrin 3.28 g/100 g, and forsythiaside 26.62 g/100 g [19]. The anti-obesity effect of 0 (control), 2.5, and 5% FLE was examined in male Sprague–Dawley (SD) rats fed an HFD. The average body weights of the groups were nearly identical at the start of the experiment, but body weight was significantly lower in the treatment groups than in the control group after four weeks of feeding as shown in Figure 3. The effect of the extract was dose-dependent. Food intake was not significantly different among the groups.

White adipose tissue (WAT) weight was dose-dependently decreased by FLE supplementation and the ratios of peritoneal WAT (WATp) and epididymal WAT (WATe) weight to body weight were also reduced by treatment. Brown adipose tissue (BAT) weight was significantly lower in the two FLE groups than in the control group, but the ratio of BAT weight to body weight was not different among the groups. These findings suggest that fat accumulation in WAT was decreased and the function for thermogenesis in BAT was invariable, giving an effective anti-obesity effect. Liver weight was significantly lower in the two FLE groups than in the control group. Plasma triglyceride (TG) and free fatty acid (FFA) levels were significantly reduced by FLE treatment as shown in Table 2.

To further explore the mechanism of the anti-obesity effect of FLE, gene expression analysis by real-time PCR on the fatty acid uptake by the liver were measured [19]. The genes were selected according to metabolic function, namely fatty acid β-oxidation, fatty acid transport, and glucose metabolism. The gene expression of fatty acid transport protein (Fatp), a regulator of fatty acid transport, was significantly higher in the two FLE groups than in the control group. The expression of carnitine palmitoyltransferase 1A (Cptla) and ACADVL, which are related to the activation of β-oxidation, was also significantly upregulated by FLE exposure as shown in Table 3. The expression of the glycolysis gene glucokinase (Gck) was only increased in the 2.5% FLE group.

These results indicated that, in HFD-fed rats, fatty acid uptake by the liver was increased by FLE, followed by an increase in fatty acid β-oxidation, which may have decreased plasma FFA levels.

Peroxisome proliferator-activated receptor ã (PPARγ) is a key transcription factor that regulates adipogenesis and lipid metabolism [20] and uncoupling protein 1 (UCP1) in BAT is a mediator of thermogenesis and regulator of lipid levels [21]. The gene expressions of PPARγ and adiponectin were significantly upregulated in WATp in the two FLE groups as shown in Table 4. The gene expression of UCP1 was significantly enhanced in BAT in the two FLE groups. Chronic administration of FLE induced PPARγ and adiponectin gene expression, which depends on the accumulation of visceral fat in WAT to improve hyperlipidemia and their upregulation might increase non-shivering thermogenesis in BAT via UCP1.

Adiponectin level in BAT was not checked as adiponectin is secreted by WAT [22].

**Figure 4:**
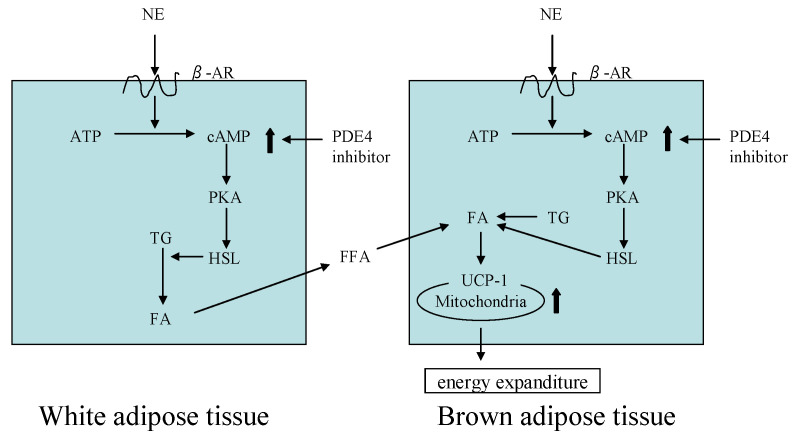
Mechanism of energy expenditure in adipose tissues induced by norepinephrine (NE). â-AR: â-Adrenergic receptor; ATP: Adenosine triphosphate; PKA; Protein kinase A; HSL; Hormone sensitive lipase; TG; Triglyceride; FA; Fatty acid; FFA: Free fatty acd, UCP1: Uncouping protein 1.

It is known that polyphenolic compounds can regulate obesity in rats fed an HFD [23]. Zhao et al. reported that phillyrin suppressed nutritive obesity in mice, including reductions of body weight, wet fat weight, the fat index, and the diameter of fat cells, and decreases of serum TG and cholesterol levels [24]. Phillyrin is a selective PDE4 inhibitor [10]. PDE4 inhibition is therapeutic strategy for metabolic disorders and results in elevated cAMP concentrations in adipose tissues as shown in Figure 4. The direct effects of cAMP on lipolysis in WAT and thermogenesis via UCP1 upregulation in BAT increase the thermogenic capacity of BAT and decrease adiposity [11].

In addition, Stephen et al. demonstrated that, in the absence of an adrenergic stimulus, a combination of two PDE inhibitors is required to fully upregulate UCP1 and increase lipolysis in BAT [25]. Thus, pinoresinol β-d-glucoside and phillyrin in forsythia leaves might be responsible for the observed anti-obesity effects. These results indicated that FLE has potential value as a health material for reducing obesity similarly as forsythia fruit in the Kampo medicine BOFU.

## 4. Combinations of Forsythia Leaves and Other Materials

BOFU, which is widely used as an anti-obesity pharmaceutical, consists of 18 crude drug components. Yoshida et al. reported that the mixture of ephedrine and extracts of glycyrrhiza, schizonepeta spikes, and forsythia fruit also exerted an anti-obesity effect corresponding to the effect of BOFU [3]. This result indicated that the combination of both the crude drugs as adrenergic stimuli and PDE inhibitors is needed to enhance the anti-obesity effect of forsythia leaves. However, most crude drugs in BOFU are barred from use in health foods in Japan. We attempted to develop a simple and beneficial anti-obesity health food by combining forsythia leaves with other commonly available herbal materials.

### 4.1. Gardenia Fruit, Glycyrrhiza, and Immature Orange

Forsythia leaves, gardenia fruit, glycyrrhiza, and immature orange (*Citrus aurantium*) were selected [26]. Forsythia leaves and glycyrrhiza contain PDE inhibitors [27]. Immature orange, which contains synephrine, is a replacement for ephedra [28]. Gardenia fruit was selected because of its reported anti-obesity effects in the literature [29].

In the first experiment, the anti-obesity effect of a mixture of herbal extracts (MHE) was investigated in SD rats fed a normal diet (ND) or HFD, both or without MHE supplementation. MHE consisted of forsythia leaf, immature orange, glycyrrhiza, and gardenia fruit extracts at a ratio of 3. 3. 5. 3. This ratio was determined by referencing the weight ratio of the four extracts in BOFU (150. 150. 250. 150 (mg/day)).

Rats fed the ND with MHE for 10 weeks exhibited decreases in WATp and WATe weight and a significant decrease in plasma TG levels compared to the findings in rats fed the ND alone as shown in Table 5. These effects of MHE were similar to those in a previous report using mice fed an ND plus BOFU [30]. The decrease of plasma TG levels was attributed to the inhibition of TG synthesis in the liver and decomposition of TG in the visceral adipose tissue by MHE. The decrease of plasma HDL-cholesterol level was exhibited in the 1% MHE group compared with the ND-control group. In a case using mice fed an ND plus 1.5% BOFU, the tendency of decrease of plasma total cholesterol value was also exhibited after three weeks [30]. So far, these reasons for the decrease are unknown.

Rats fed the HFD containing 5% MHE for 10 weeks exhibited significant decreases in body, WATp, and WATe weight and significant decreases in plasma TG, FFA, total cholesterol, glucose, and insulin levels compared to the results in the HFD control group as shown in Table 6. In addition, the 5% MHE group exhibited a marked increase in plasma adiponectin levels under the HFD condition. These effects in the 5% MHE group were similar to those in the previous reports that used mice who were fed an HFD containing BOFU [3,31,32].

A study by Hioki et al. indicated that BOFU improved visceral adiposity and insulin resistance in obese women with impaired glucose tolerance [33]. Therefore, the results of this experiment suggested that the fat mass-lowering effect of MHE may be attributable to a direct pharmacological action on adipose tissues similar to that of BOFU.

**Figure 5:**
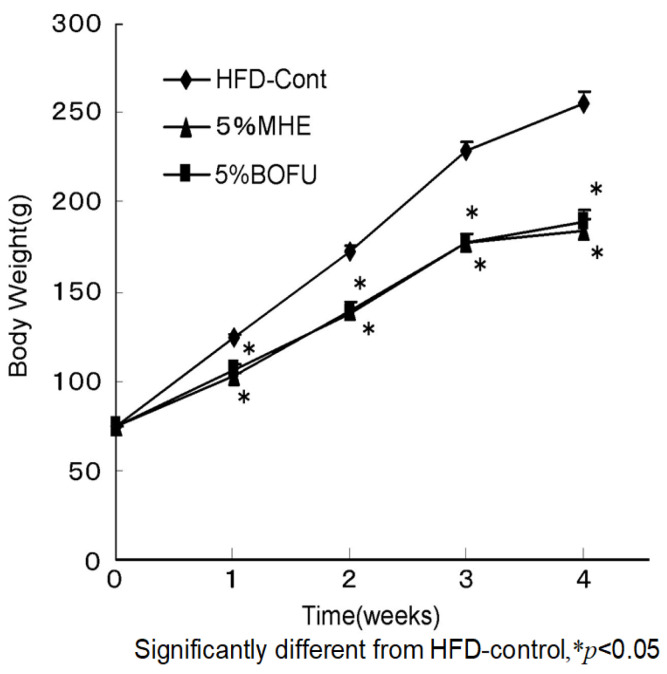
Effects of MHE and BOFU on body weight in HFD-fed rats.

In the second experiment, body, WATp, WATe, and BAT weight were compared in HFD-fed rats treated with 5% MHE or 5% BOFU (ALPS Pharmaceutical Ind. Co.) as shown in Figure 5 and Table 7. Real-time PCR demonstrated that fatty acid uptake by the liver was increased by MHE and BOFU in HFD-fed rats, followed by an increase in fatty acid β-oxidation as shown in Table 8.

To compare the anti-obesity effects among 5% MHE and 5% BOFU groups, rats were divided into three groups (0% control, 5% MHE, and 5% BOFU groups) based on their body weight. Gene expressions by fold change to control in 5% MHE and 5% BOFU groups were calculated with that of the control group as value of 1, respectively.

Obesity is a common risk factor for type 2 diabetes. Adiponectin gene expression and plasma adiponectin levels were reported to be significantly reduced in obese/diabetic mice and humans [34].

Under HFD conditions, chronic administration of MHE or BOFU induced the gene expression of PPARγ and adiponectin as shown in Table 9. These effects were considered to result in the observed increases of plasma adiponectin levels, decreases of visceral fat accumulation in WATp and WATe and reductions of plasma glucose levels in the first experiment, thereby improving insulin resistance. That is, PPARγ gene expression induces an increase of plasma adiponectin levels [35]. In addition, Matsuzawa et al. reported that plasma adiponectin levels decrease with visceral fat accumulation [36]. This finding indicates that the increase of plasma adiponectin levels is related to the decrease of visceral fat accumulation.

It is noteworthy that UCP1 gene expression in BAT was significantly upregulated in the MHE group. UCP1 expression in BAT is known as a significant component of whole body energy expenditure and its dysfunction contributes to the development of obesity [37]. These findings revealed that MHE or BOFU supplementation inhibited visceral fat accumulation in HFD-fed rats, and both supplements upregulated the UCP1 gene, which regulates energy metabolism in WAT. These results suggested that MHE exerts similar anti-obesity effects as BOFU. It can be presumed that gardenoside and geniposide in gardenia fruit, synephrine in immature orange, pinoresinol β-d-glucoside and phillyrin in forsythia leaves, and glabridin in glycyrrhiza are partly responsible for the synergistic effects of MHE, supporting the use of the extract as a simple and beneficial supplement for preventing diet-induced obesity.

### 4.2. Eucommia Leaves

We expected that eucommia leaves would synergistically enhance the effects of forsythia leaves. The leaves of *Eucommia ulmoides* Oliv. have been used in the health tea “Tochucha” for anti-hypertensive purposes in Japan [38]. The main constituents of the leaves are iridoid glucosides, asperuloside, and geniposidic acid, which have similar chemical structures as iridoid glucosides, gardenoside, and geniposide in gardenia fruit, respectively [39].

We reported the anti-obesity effects in eucommia leaf extract (ELE) [35] in comparison with asperuloside [40]. ELE was comprised of asperuloside 13.7 mg/g and geniposidic acid 69.5 mg/g. As presented in Table 10 and Table 11, the oral administration of ELE or asperuloside to HFD-fed rats resulted in anti-obesity effects, confirming that asperuloside is one of the bioactive compounds responsible for the anti-obesity effects of ELE.

**Figure 6:**
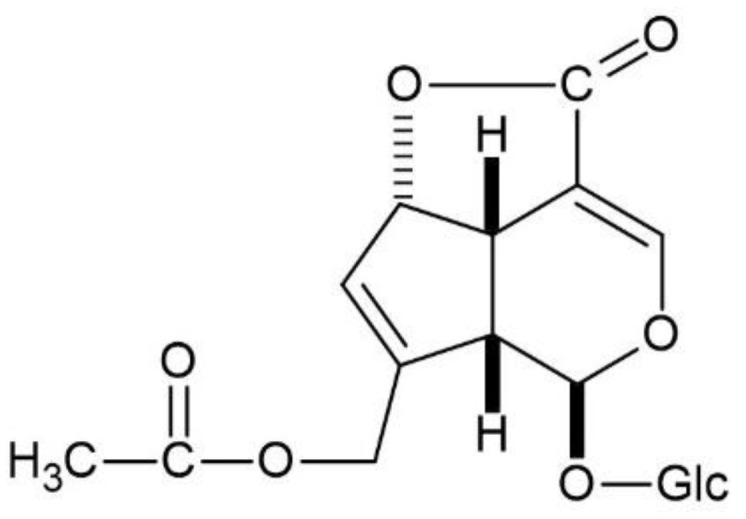
Chemical structure of asperuloside.

We revealed for the first time that oral asperuloside administration to rats increased the bile acid pool in the intestine [38]. Gardenoside from gardenia fruit is known to protect the leaves against insect feeding. Regarding the mechanism, it was demonstrated that gardenoside is easily hydrolyzed to aglycone in the digestive tract by β-glucosidase, followed by its binding with proteins of transporters to cause lethal damage (Figure 6) [41].

It has long been known that the leaves of *Eucommia* are hardly damaged by insects. This suggests that the leaves contain the protective compound. In the same manner, asperuloside is easily hydrolyzed to aglycone by β-glucosidase. It has been reported that aglycone binds with amino acids to produce resinous substances in vitro similarly as gardenoside [42]. Meanwhile, asperuloside plasma levels are extremely low in rats (Cmax = 198 ng/mL at 50 mg/kg p.o., unpublished data). In addition, a review article stated that metformin increases the bile acid pool within the intestine, predominantly through reduced ileal absorption [43]. Therefore, it is assumed that the increase of the bile acid pool in the intestine following asperuloside administration in rats is also attributable to the reduction of ileal absorption of bile acid by the transporter bound by the aglycone of asperuloside. Further research is required to clarify the mechanism.

**Figure 7:**
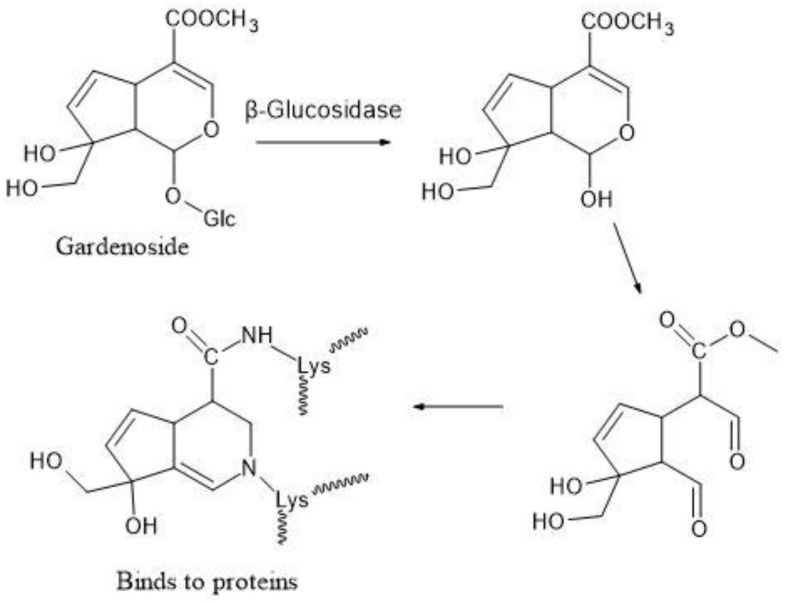
Mechanism by which gardenoside induces binding with proteins of transporters.

Prior research recorded increases of the bile acid pool in the intestine and cholic acid levels in plasma after oral cholic acid treatment in HFD-fed mice [44]. An increased plasma LDL level is also observed, following increases of the bile acid pool in the intestine [45]. The increasing cholic acid content in the blood acts on TGR5 receptors of BAT in rodents and muscle skeletal in humans to exert its anti-obesity effect (Figure 7) [46]. Therefore, it is assumed that the anti-obesity effect of asperuloside in rodents might depend on an indirect effect of cholic acid, as opposed to a direct effect of asperuloside.

Furthermore, the increase of the bile acid pool following metformin was accompanied by a marked increase of *Akkermansia muciniphila* counts in the gut gastrointestinal tract [43], and similar increases in *A. muciniphila* counts in the gastrointestinal tract were induced by asperuloside [47]. In addition, it was recently reported that BOFU containing gardenoside from gardenia fruit can induce marked increases of *A. muciniphila* counts in the gastrointestinal tract [48]. *A. muciniphila* is expected to emerge as a beneficial microbe, which produces active short chain fatty acids for anti-obesity effects from the dietary fiber in the gut [49].

**Figure 8:**
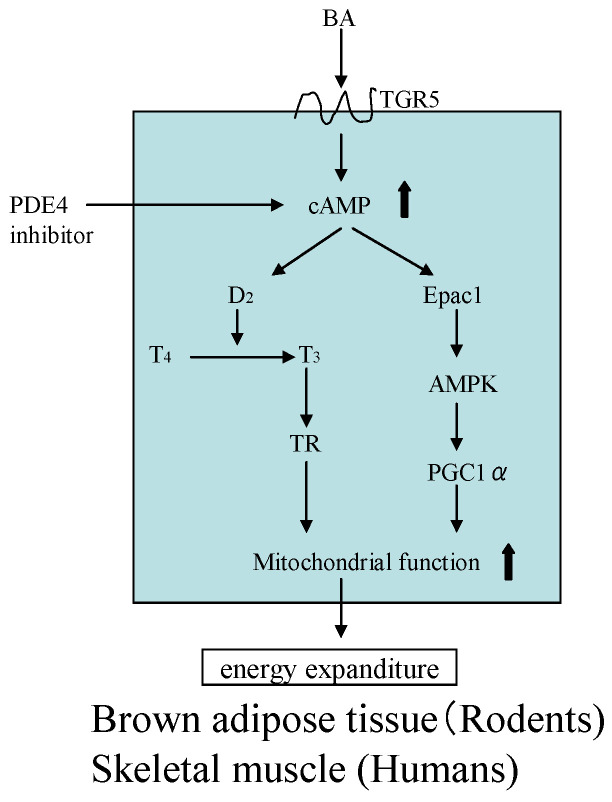
Mechanism of energy expenditure induced by bile acid (BA) via brown adipose tissue or skeletal muscle. TGR5: G protein-coupled receptor 5; D: Iodothyronine deiodinase; T: Thyroxine; TR: Thyroido hormone receptor; Epac 1: Exchange protein activated by cyclic AMP 1; AMPK: AMP-activated kinase; PGC 1á; Peroxisome proliferators-activated-receptor ã co-activator-1á.

Conversely, the anti-obesity effects of the ELE product were weak in humans unlike the case of rodents [50]. This finding is believed to be associated with the lack of BAT activation by cholic acid via TGR5 in humans [51]. Thermogenesis in skeletal muscle is low in humans compared to that in BAT (skeletal muscle:BAT = 1:100) [52]. A prior report indicated that mitochondrial activity in skeletal muscle is enhanced by PDE4 inhibition as shown in Figure 8 [53].

BOFU contains the active iridoid glucosides, gardenoside and geniposide from gardenia fruit, and polyphenol, phillyrin from forsythia fruit. Similarly, eucommia leaves contain the active iridoid glucosides, asperuloside, and geniposidic acid, and forsythia leaves contain polyphenol, phillyrin.

From the aforementioned results, the simple combination of forsythia leaves and eucommia leaves as an herbal tea are expected to have synergistic effects on obesity in humans.

## 5. Other Biological Activities

### 5.1. Effects for Forsythia Leaves on Atopic Dermatitis

It has been known for more than 40 years that high numbers of *Staphylococcus aureus* grow when samples from the skin of patients with atopic dermatitis are cultured. It has long been debated whether *S. aureus*, which grows on the skin, is the cause of inflammation or the result of chronic inflammation. Kobayashi et al. revealed using ADAM17 cKO mice that when atopic dermatitis becomes severe, the diversity of bacteria on the skin surface is significantly reduced, and *S. aureus* becomes dominant [54]. A causal relationship between atopic dermatitis and *S. aureus* has not been established to date because of the lack of a suitable animal model.

Since colonization by *S. aureus* is the cause of inflammation in atopic dermatitis, it is suggested that a component with antibacterial activity against *S. aureus* could suppress inflammation. We previously reported that forsythia extract (MIC = 6% *w/v*) and its main constituent forsythiaside (MIC = 3.2 mM) have strong antibacterial effects against *S. aureus* Terashima [55,56]. Qu et al. evaluated the anti-microbial activity of forsythiaside and phillyrin against *Escherichia coli*-10B, *Pseudomonas aeruginosa* and *S. aureus* Rn4220 using the microtiter plate method. Forsythiaside exhibited strong antibacterial activity against all three bacteria, and it was more effective against *S. aureus* (MIC = 76.67 μg/mL) than tetracycline (MIC = 119.05 μg/mL). However, the activity of phillyrin was not remarkable [57]. These results support the use of forsythia as an herbal medicine for skin diseases.

Furthermore, Sung et al. evaluated the in vivo and in vitro therapeutic effects of *Forsythia suspensa* fruit extract (FSE) in an NC/Nga mouse model exposed to *Dermatophagoides farinae* crude extract (DfE). Topical application of FSE on lesional skin of DfE-induced atopic dermatitis mice effectively alleviated the development of atopic dermatitis-like lesions by suppressing the expression of chemokines, cytokines, and adhesion molecules in keratinocytes. In addition, among the components of FSE, forsythiaside and phillyrin inhibited the production of thymus- and activation-regulated chemokine (TARC), macrophage-derived chemokine (MDC), and regulated activation, normal T cell expressed, and secreted (RANTES) in human keratinocytes [12]. These results suggested that forsythia fruit or leaves might be also useful candidate for treating allergic skin inflammatory disorders.

Ahluwalia et al. summarized the effects of PDE4 inhibitors on atopic dermatitis in a review article [58]. In particular, increased PDE4 activity is correlated with inflammatory dysregulation in patients with atopic dermatitis [59,60,61]. Increased PDE4 activity was also detected in cord blood cells from newborns with atopic parents. leading to speculation about a genetic abnormality [61].

Multiple in vitro assays have revealed decreased production of PGE2, IL-4, and IL-10 in atopic leukocytes exposed to high-potency PDE4 inhibitors [62]. These predictions were later confirmed in vivo by the finding that PDE4 inhibitors reduced cytokine and mediator release, either by directly acting on IL-4 production or the Th2 cell balance [59]. This clinical anti-inflammatory effect serves as the rationale for developing PDE4 inhibitors for atopic dermatitis. Currently, several PDE4 inhibitors including topical and oral formulation, have been developed to target the inflammatory cascade of atopic dermatitis [58].

**Figure 9:**
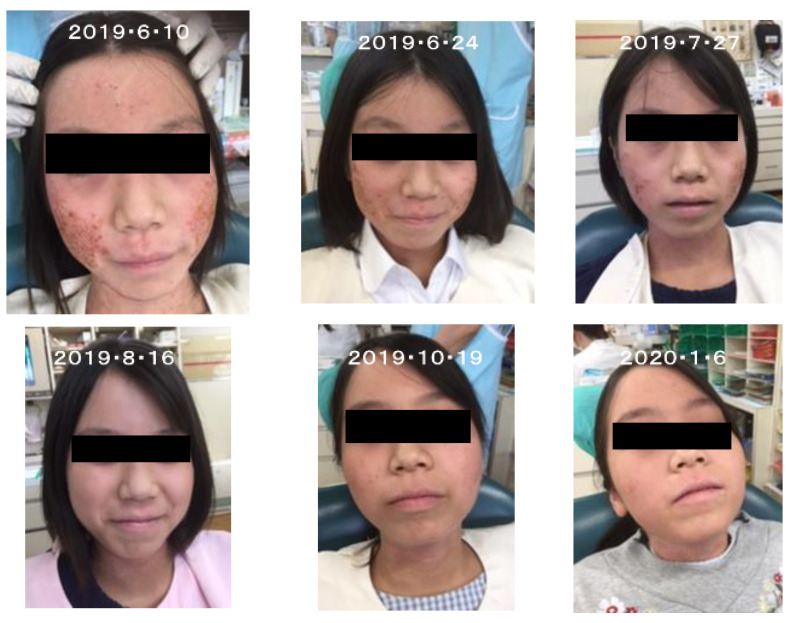
Improvement of atopic dermatitis by the treatment with forsythia leaf decoction between 10 June 2019 and 6 January 2020 (photos sent from Dr. O. Katayama).

From these results, forsythia leaves are expected to have efficacy against atopic dermatitis as indicated in the clinical photos sent from Dr. O. Katayama (Figure 9).

### 5.2. Effects of Forsythia Leaves against Influenza A Virus Infection

Deng et al. reported that forsythiaside controlled influenza A virus infection and improved the course after infection in SPF C57BL/6j mice [14]. Specifically, body weight was reduced by virus infection, and this change was alleviated in forsythiaside and oseltamivir-administered mice. The drugs also suppressed pathological damage in the lungs and reversed the upregulation of TLR7 mRNA expression. These findings suggested that forsythiaside can improve the outcome of influenza A virus infection by inhibiting replication of the virus.

The anti-viral effect of phillyrin against influenza A virus infection in vivo was investigated to identify a novel anti-viral drug [13]. The administration of phillyrin at a dose of 20 mg/kg/day for three days significantly prolonged the mean survival time, reduced the lung index, decreased viral titers and IL-6 levels, reduced lung hemagglutinin levels, and attenuated lung tissue damage in mice infected with influenza A virus. PDE4 inhibition has been demonstrated to ameliorate acute lung injury caused by influenza A virus in mice [10]. Thus, PDE4 inhibitors such as phillyrin may specifically ameliorate airway and lung inflammation.

From these reports, forsythia leaves are expected to have effects against influenza A virus infection. Table 12 presents the results of a preliminary test of the preventive effects of forsythia leaf tea against influenza A virus in a Japanese middle school between December 2018 and February 2019. The experiment indicated that forsythia leaf tea may be effective for preventing influenza virus infection. More detailed intake tests are needed in the future.

### 5.3. Function of Phillyrin as a Phytoestrogen

Phytoestrogens, also termed plant estrogens, are exogenous estrogens that have the same function as female hormones. As the major phytoestrogens, soy isoflavone, its intestinal bacterial metabolite, equol, and enterolactone, a plant lignan metabolite generated by intestinal bacteria, have been described. These compounds have estrogen-like activity, and they are expected to have effects such as preventing breast cancer, menopause and osteoporosis in women and thyroid cancer in men [63].

**Figure 10:**
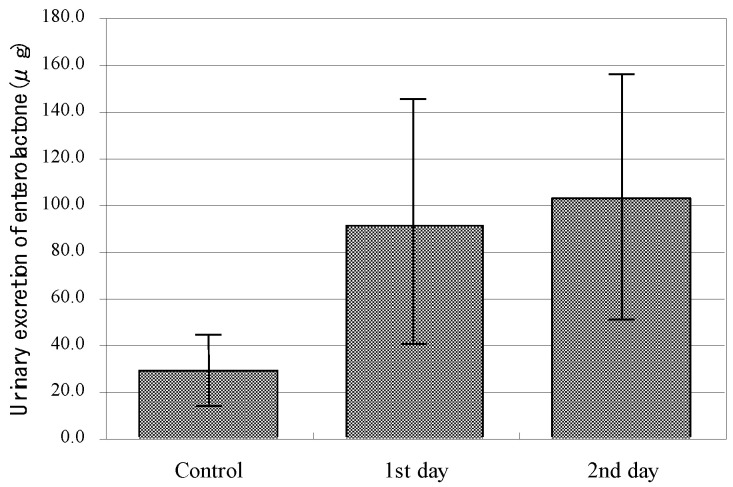
Urine excretion of enterolactone (µg) in male SD rats following the oral administration of phillyrin (25 mg/kg) (data from Prof. H. Adlercreutz).

**Figure 11:**
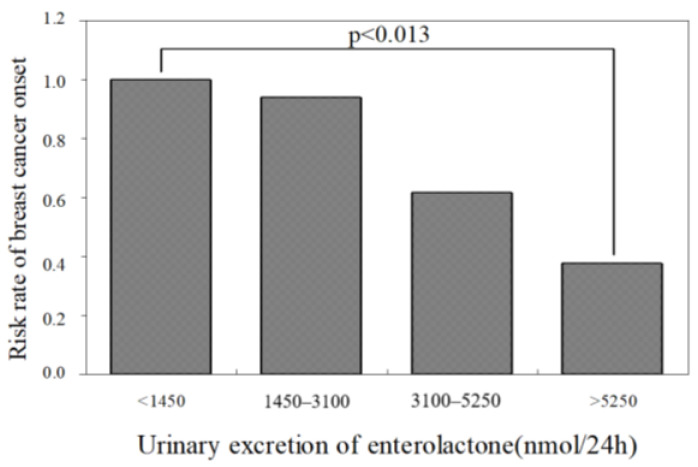
Breast cancer risk and urinary excretion of enterolactone in 144 subjects (data from Prof. H. Adlercreutz).

**Figure 12:**
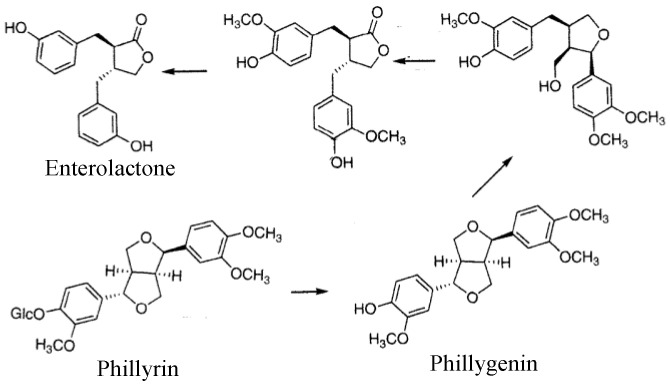
Estimated pathway of the metabolism of phillyrin to enterolactone by intestinal bacteria.

We conducted joint research on metabolites of lignans produced by gastrointestinal flora [6,64], confirming that enterolactone was excreted in urine when phillyrin was orally administered to male SD rats as shown in Figure 10. The results demonstrated that the risk of breast cancer was decreased as shown in Figure 11 and menopausal symptoms were suppressed as the urinary excretion of enterolactone increases in humans [65]. It was clarified that phillyrin is metabolized by intestinal bacteria to the phytoestrogen enterolactone. The estimated metabolic pathway by intestinal bacteria is presented in Figure 12 [6].

Based on these findings, forsythia leaves are expected to be beneficial materials based on the phytoestrogen content.

## 6. Toxicity and Safety of Forsythia Leaves

We examined the toxicity of FLE following a single oral dose and repeated administration for 14 days in male ddY mice [66]. Following a single oral dose of as much as 6.67 g/kg/day FLE (equivalent to 18.4 g/kg forsythia leaves), some mice displayed slight sedation and subsequent excitement immediately after administration, but this effect was not observed on subsequent days. The treatment did not influence body weight as shown in Table 13.

Repeated oral administration of 0.166 g/kg/day FLE (0.46 g/kg equivalent to forsythia leaves) for 14 days did not cause any obvious changes to general symptoms, body weight, and organ weight as shown in Table 14.

There were no deaths in mice that received a single dose, and the LD_50_ of FLE was estimated to be at least 10 g/kg or more, suggesting that its toxicity was low at the moment.

Prior research identified no acute toxicity of FLE or the ethanol extract of forsythia leaves in mice, even at a daily dose of 61.60 g/kg [67]. Conversely, forsythiaside displayed cytotoxicity in PK-15, Mark-145, and CHK cells with IC_50_ values of 0.138, 0.087, and 0.384 mg/mL, respectively [68] and also caused acute toxicity in mice (IC_50_ = 1.98 g/kg) [69].

Han et al. reported the toxicity and safety of phillyrin, following a single oral dose of 18,100 mg/kg in NIH mice [70]. Mortality was not observed after 14 days, and no clinically relevant adverse effects or variations in body weight or food consumption were observed. The maximum tolerated dose of phillyrin was determined to exceed 18,100 mg/kg. Furthermore, sub-chronic toxicity was evaluated, following the oral administration of 0, 540, 1620, and 6480 mg/kg phillyrin for 30 days in SD rats. After 30 days, no mortality or significant toxicological effects such as decreased food consumption, body weight, or changes of biochemical parameters in serum and vital signs were observed. The no-observed-adverse-effect level after 30 days was 6480 mg/kg body weight. These results indicated that the oral phillyrin has low or no toxicity.

The additional biochemical and histopathological examinations will be required for further evaluation of the toxicity and safety of forsythia leaves by us.

Regarding human intake, no adverse events have been reported for forsythia leaf tea even though it has been on the market in Niigata Prefecture, Japan for more than 17 years.

## 7. Limitations of the Study

In this review, we demonstrated that forsythia leaves have potential value as a health material for reducing obesity in SD rats fed an HFD. However, anti-obesity effects in humans are still to be determined.

## 8. Conclusions

Forsythia fruit, the dried fruit of *F. suspensa*, is listed in Japanese, Chinese, and Korean Pharmacopoeias. Up to now, many compounds have been identified in the fruit. The major compounds are phillyrin, pinoresinol α-d-glucoside, and forsythiaside. Many pharmacological studies have confirmed that forsythia fruit possesses anti-inflammatory, antioxidant, antiviral, antivomiting, and antitumor activities, as well as hepatoprotective, neuroprotective, and cardiovascular protective effects [71].

Forsythia fruit is used in many Kampo medicines in Japan as a principal drug. However, there are a number of factors that limit the wide use of the forsythia fruit. Fruits cannot be harvested in Japan because *F. suspensa* growing in Japan does not have fruits. Forsythia fruit used in Kampo medicines is imported from the People’s Republic of China. In addition, the use of forsythia fruit as a health food is prohibited in Japan.

We found that the major compounds in forsythia leaves are also phillyrin, pinoresinol α-d-glucoside, and forsythiaside, similar to those of forsythia fruit [5,6]. Therefore, we studied the biological effects of forsythia leaves as an alternative to forsythia fruit.

In particular, phillyrin was reported as a novel selective PDE4 inhibitor [10]. PDE4 inhibition is a therapeutic strategy for metabolic disorders [11]. Increased PDE4 activity is correlated with inflammatory dysregulation in patients with atopic dermatitis [59,60,61].

Furthermore, PDE4 inhibition has been demonstrated to ameliorate acute lung injury caused by influenza A virus in mice [10]. We reported that phillyrin is metabolized to enterolactone as phytoestrogen by gastrointestinal flora.

In this review, we summarized our studies on the biological effects of forsythia leaves containing phillyrin and other polyphenolic compounds, particulary against obesity, atopic dermatitis, and influenza A virus infection, and its potential as a phytoestrogen.

In conclusion, forsythia leaves were revealed to be useful and safe as a health food containing a PDE4 inhibitor, supporting its use in the treatments of metabolic disorders and inflammatory dysregulation. Furthermore, we expect that forsythia leaves will also be used as one of the new drug sources in the future.

## Figures and Tables

**Table 1:** Chemical shifts for C-1′ and C-1″ of phillyrin and phillygenin derivatives.

	C-1′	C-1″
Phillyrin	131.0	135.4
Phillygenin methyl ether	131.0	133.8
Phillygenin	131.0	133.1
Phillygenin acetate	131.0	140.3

**Table 2:** Effects of FLE on physical and plasma parameters after four weeks of HFD feeding.

	Diet		
	Control (*n* = 9)	2.5% FLE (*n* = 8)	5.0% FLE (*n* = 8)
Food intake (g/rat/day)			
The first week	13.3 ± 0.25	12.6 ± 0.61	12.9 ± 0.26
The fourth week	15.6 ± 0.71	14.9 ± 0.88	14.4 ± 073
Body weight and organ weight (g/rat)			
Initial body weight	74.7 ± 0.58	75.3 ± 1.40	74.8 ± 0.91
Final body weight	255.1 ± 7.41	167.8 ± 1.77 *	148.2 ± 3.74 *
Perirenal white adipose tissue	1.44 ± 0.22	0.47 ± 0.10 *	0.33 ± 0.07 *
Epididymal white adipose tissue	3.14 ± 0.21	1.22 ± 0.07 *	1.02 ± 0.09 *
Brown adipose tissue	1.02 ± 0.04	0.61 ± 0.02 *	0.59 ± 0.03 *
Liver	2.05 ± 0.05	1.55 ± 0.09 *	1.51 ± 0.06 *
Plasma parameters			
Triglyceride (mg/dL)	124.4 ± 15.1	65.5 ± 11.4 *	47.4 ± 4.7 *
Free fatty acid (μEq/L)	474.3 ± 29.5	318.8 ± 35.4 *	280 ± 18.6 *
HDL-cholesterol (mg/dL)	38.0 ± 1.3	46.1 ± 2.4	47.8 ± 1.6
LDL-cholesterol (mg/dL)	14.0 ± 0.8	17.0 ± 0.9	15.4 ± 0.7
Total cholesterol (mg/dL)	102.6 ± 3.7	121.9 ± 5.0	115 ± 3.9
Glucose (mg/dL)	168.4 ± 4.3	174.8 ± 16.6	168.1 ± 4.9

Each value represents the mean ± S.E. Sinificantly different from Control: * *p* < 0.05.

**Table 3:** Gene expression analysis by real-time PCR in the liver after four weeks of HFD feeding.

	Fold Change to Control	
Gene Name (Accession No)	2.5% FLE	5.0% FLE
Glycolitic system		
Gck (NM012565)	1.66 ± 0.14 *	1.53 ± 0.05
TCA cycle		
Cs (NM130755)	1.03 ± 0.07	1.21 ± 0.10
Ogdh (AI412142)	0.89 ± 0.05	0.96 ± 0.04
Electron transfer system		
Comp. I (CB5449004)	0.99 ± 0.02	1.03 ± 0.07
Comp. IV (NM017202)	1.84 ± 0.07 *	1.98 ± 0.05 *
Fatty acid synthesis		
Fasn (NM017332)	1.19 ± 0.13	1.24 ± 0.08
Fatty acid transporter		
Fatp (NM053580)	1.91 ± 0.11 *	1.92 ± 0.04 *
Fatty acid β-oxidation		
Cpt1o (NM031559)	1.84 ± 0.03 *	1.81 ± 0.15 *
Acadvl (NM012891)	1.94 ± 0.67 *	1.91 ± 0.10 *

Each value represents the mean ± S.E. (*n* = 6). Sinificantly different from Control: * *p* < 0.05.

**Table 4:** Gene expression analysis in adipose tissue by real-time PCR after four weeks of HFD feeding.

	Fold Change to Control	
Gene Name (Accession No)	2.5% FLE	5.0% FLE
Perirenal white adipose tissue		
Fatty acid receptor and adipocytokine		
PPARγ (NM013124)	2.74 ± 0.04 **	2.85 ± 0.13 **
Adiponectin (NM144744)	1.93 ± 0.13 *	1.97 ± 0.03 *
Resistin (AJ555618)	1.56 ± 0.10	1.33 ± 0.13
Brown adipose tissue		
Uncopling ATP synthesis from oxidative metabolism		
UCP1 (NM012682)	1.88 ± 0.02 *	1.92 ± 0.11 *

Each value represents the mean ± S.E. (*n* = 6). Signicantly different from Control: * *p* < 0.05, ** *p* < 0.01.

**Table 5:** Effects of MHE on physical and plasma parameters in ND-fed rats after 10 weeks.

		Diet (ND)		
	Control (*n* = 4)	0.2% MHE (*n* = 4)	1% MHE (*n* = 4)	5% MHE (*n* = 4)
Food intake (g/rat)				
Final day	43.2 ± 1.2	44.1 ± 0.3	40.2 ± 0.8	44.2 ± 0.1
Body weight (g/rat)				
Final body weight	491 ± 13	445 ± 14	454 ± 16	416 ± 15
Organ weight/(g/100 g body weight)				
Perirenal white adipose tissue	0.67 ± 0.16	0.36 ± 0.06	0.36 ± 0.06	0.31 ± 0.05 *
Epididymal white adipose tissue	1.71 ± 0.21	1.14 ± 0.14 *	1.29 ± 0.13	1.03 ± 0.06 *
Brown adipose tissue	0.13 ± 0.01	0.15 ± 0.01	0.18 ± 0.01	0.19 ± 0.02
Plasma parameters				
Triglyceride (mg/dL)	232 ± 38	128 ± 19 *	89.0 ± 3.8 *	81.5 ± 4.0 *
Free fatty acid (μEq/L)	355 ± 46	362 ± 30	262 ± 22	313 ± 68
HDL-cholesterol (mg/dL)	32.0 ± 1.7	27.3 ± 1.1	25.8 ± 1.2 *	31.5 ± 2.2
LDL-cholesterol (mg/dL)	11.0 ± 0.7	8.50 ± 0.29	8.50 ± 0.87	8.25 ± 0.25
Total cholesterol (mg/dL)	93.5 ± 7.7	72.0 ± 2.3	68.0 ± 4.2 *	81.5 ± 5.8
Glucose (mg/dL)	150 ± 7	151 ± 3	138 ± 5	154 ± 4
Insulin (ng/mL)	1.27 ± 0.31	1.49 ± 0.06	1.49 ± 0.23	1.90 ± 0.15
Adiponectin (ng/mL)	3881 ± 461	4749 ± 1111	3223 ± 360	3717 ± 544

Each value represents the mean ± S.E. Significantly different from ND-control: * *p* < 0.05.

**Table 6:** Effects of MHE on physical and plasma parameters in HFD-fed rats after 10 weeks.

		Diet (HFD)		
	Control (*n* = 6)	0.2% MHE (*n* = 6)	1% MHE (*n* = 6)	5% MHE (*n* = 6)
Food intake (g/rat)				
Final day	35.5 ± 0.4	31.8 ± 0.8	32.9 ± 0.5	39.3 ± 3.7
Body weight (g/rat)				
Final body weight	588 ± 6 ^†^	561 ± 23	539 ± 13	410 ± 5 *
Organ weight (g/100g body weight)				
Perirenal white adipose tissue	1.64 ± 0.06 ^†^	1.42 ± 0.11	1.40 ± 0.15	0.74 ± 0.11 **
Epididymal white adipose tissue	3.41 ± 0.07 ^†^	3.03 ± 0.22	2.53 ± 0.10 *	1.53 ± 0.17 **
Brown adipose tissue	0.20 ± 0.01 ^†^	0.21 ± 0.02	0.28 ± 0.02	0.24 ± 0.02
Plasma parameters				
Triglyceride (mg/dL)	229 ± 6	164 ± 36	207 ± 47	65.3 ± 16.4 **
Free fatty acid (μEq/L)	530 ± 25 ^†^	387 ± 5 *	396 ± 26 *	454 ± 51
HDL-cholesterol (mg/dL)	32.2 ± 2.5	28.7 ± 1.2	34.0 ± 2.1	40.7 ± 1.3 *
LDL-cholesterol (mg/dL)	15.7 ± 1.4 ^†^	11.3 ± 1.0 *	14.3 ± 1.2	13.7 ± 1.7
Total cholesterol (mg/dL)	125 ± 7 ^†^	83.0 ± 2.5 *	101 ± 10 *	110 ± 7
Glucose (mg/dL)	177 ± 5 ^†^	160 ± 6 *	162 ± 3 *	153 ± 3 *
Insulin (ng/mL)	2.62 ± 0.07 ^†^	1.00 ± 0.13 **	1.49 ± 0.30 *	1.31 ± 0.28 *
Adiponectin (ng/mL)	3332 ± 200	6342 ± 998 *	4079 ± 237	5194 ± 757 *

Each value represents the mean ± S.E. Significantly different from HFD-control: * *p* < 0.05, ** *p* < 0.01. Significantly different from ND-control: ^†^ *p* < 0.05.

**Table 7:** Effects of MHE and BOFU on body and organ weight after four weeks of feeding.

	Diet (HFD)		
	Control (*n* = 9)	5% MHE (*n* = 8)	5% BOFU (*n* = 8)
Body weight (g/rat)			
Initial body weight	74.7 ± 0.6	74.8 ± 0.9	74.2 ± 0.9
Final body weight	255.1 ± 7.4	183.3 ± 7.8 *	189.6 ± 5.9 *
Organ weight (g/100 g body weight)			
Perirenal white adipose tissue	0.53 ± 0.08	0.31 ± 0.08 *	0.31 ± 0.07 *
Epididymal white adipose tissue	1.2 ± 0.06	0.63 ± 0.09 *	0.69 ± 0.05 *
Brown adipose tissue	0.38 ± 0.01	0.35 ± 0.02	0.36 ± 0.02

Each value represents the mean ± S.E. Significantly different from Control: * *p* < 0.05.

**Table 8:** Liver gene expression analysis by real-time PCR after four weeks of MHE and BOFU supplementation.

	Fold Change to Control	
Gene Name (Accession No)	5% MHE	5% BOFU
Glycolytic system		
Gck (NM012565)	0.54 ± 0.05 ***	0.45 ± 0.09 **
TCA cycle		
Cs (NM130755)	1.08 ± 0.12	0.97 ± 0.09
Ogdh (AI412142)	1.16 ± 0.06	1.18 ± 0.06
Electron transfer system		
Comp. I (CB5449004)	1.49 ± 0.06	0.89 ± 0.06
Comp. IV (NM017202)	1.89 ± 0.04 *	1.84 ± 0.07 *
Fatty acid synthesis		
Fasn (NM017332)	0.46 ± 0.03 ***	0.80 ± 0.11
Fatty acid transporter		
Fatp (NM053580)	1.87 ± 0.20 *	1.82 ± 0.17 *
Fatty acid β-oxidation		
Cpt1α (NM031559)	1.94 ± 0.06 *	1.74 ± 0.06 *
ACADVL (NM012891)	1.87 ± 0.11 *	1.87 ± 0.09 *

Each value represents the mean ± S.E. (*n* = 6). Significantly different from Control: * *p* < 0.05, ** *p* < 0.01, *** *p* < 0.001.

**Table 9:** Gene expression analysis in adipose tissue by real-time PCR following four weeks of MHE and BOFU exposure.

	Fold Change to Control	
Gene Name (Accession No)	5% MHE	5% BOFU
Perirenal white adipose tissue		
Fatty acid receptor and adipocytokine		
PPARγ (NM013124)	2.22 ± 0.09 *	1.89 ± 0.25 *
Adiponectin (NM144744)	2.42 ± 0.05 **	1.92 ± 0.19 *
Resistin (AJ555618)	1.30 ± 0.07	1.60 ± 0.17
Brown adipose tissue		
Uncoupling ATP synthesis from oxidative metabolism		
UCP1 (NM012682)	1.86 ± 0.09 *	1.32 ± 0.05

Each value represents the mean ± S.E. (*n* = 6). Significantly different from Control: * *p* < 0.05, ** *p* < 0.01.

**Table 10:** Effects of ELE on physical and plasma parameters in HFD-fed rats after three months.

	Diet (HFD)		
	Control (*n* = 8)	3% ELE (*n* = 8)	9% ELE (*n* = 8)
Final body weight (g/rat)	548.6 ± 16.8	485.3 ± 13.6 *	422.2 ± 17.7 *
Food intake (g/day/rat)	25.3 ± 3.0	19.7 ± 3.6	15.2 ± 1.7 *
WAT weight (g/rat)			
Perirenal white adipose tissue	10.0 ± 0.8	5.6 ± 0.4 ***	3.5 ± 0.6 ***
Epididymal white adipose tissue	18.3 ± 0.7	13.9 ± 0.6 ***	5.8 ± 0.4 ***
Plasma parameters			
Glucose (mg/L)	1520 ± 17	1458 ± 5 *	1433 ± 17 *
Insulin (ng/mL)	6.6 ± 0.5	4.2 ± 0.5 **	2.4 ± 0.3 ***
Free fatty acid (µEq/L)	610.4 ± 78.8	450.8 ± 33.8	493.4 ± 26.2
Total cholesterol (mg/L)	780 ± 27	655 ± 28 **	725 ± 15
Adiponectin (µg/L)	27 ± 3	42 ± 4	53 ± 4 **
TNF-α (pg/mL)	178.5 ± 22.6	137.1 ± 15.1	63.5 ± 8.3 *
Resistin (ng/mL)	187.6 ± 15.9	175.9 ± 15.9	111.4 ± 11.0 **
Leptin (ng/mL)	6.8 ± 0.4	5.9 ± 0.7	6.7 ± 0.8

Each value represents the mean ± S.E. Significantly different from HFD-control: * *p* < 0.05, ** *p* < 0.01, *** *p* < 0.001(Tukey HSD).

**Table 11:** Effects of ASP on physical and plasma parameters in HFD-fed rats after three months.

	Diet (HFD)			
	Control (*n* = 6)	0.03% ASP (*n* = 6)	0.1% ASP (*n* = 6)	0.3% ASP (*n* = 6)
Initial body weight (g/rat)	71 ± 1.0	71.2 ± 1.5	72.5 ± 0.5	71 ± 0.6
Food intake (g/day/rat)	27.8 ± 2.2	21.3 ± 3.2 *	17.7 ± 2.7 *	14.9 ± 2.0 *
Final body weight (g/rat)	564 ± 9	516 ± 19 *	465 ± 8 *	461 ± 7 *
Body weight gain (g/rat)	493 ± 10	445 ± 18 *	393 ± 8 *	390 ± 7 *
Relative WAT weight (%)				
Perirenal white adipose tissue	2.7 ± 0.3	1.5 ± 0.2 *	1.4 ± 0.1 *	1.3 ± 0.1 *
Epididymal white adipose tissue	2.6 ± 0.2	2.5 ± 0.2	2.2 ± 0.1	2.0 ± 0.1
Relative BAT weight (%)	0.24 ± 0.02	0.31 ± 0.01 *	0.33 ± 0.02 *	0.37 ± 0.02 *
Relative Sol. M. weight (%)	0.07 ± 0.01	0.07 ± 0.01	0.07 ± 0.01	0.07 ± 0.01
Plasma parameters				
Glucose (mg/L)	1621 ± 71	1501 ± 37 *	1394 ± 42 *	1338 ± 55 *
Insulin (ng/mL)	7.7 ± 0.6	5.2 ± 1.1 *	3.9 ± 0.8 *	3.3 ± 0.6 *
Free fatty acid (µEq/L)	639.1 ± 33.7	449 ± 56.0 *	402.7 ± 21.6 *	397.3 ± 20.9 *
Total cholesterol (mg/L)	880 ± 34	721 ± 25 *	708 ± 24 *	664 ± 26 *
Adiponectin (µg/L)	29 ± 5	39 ± 6 *	48 ± 4 *	53 ± 3 *
TNF-α (pg/mL)	198.3 ± 18.2	136.5 ± 13.1 *	98.7 ± 9.2 *	70.6 ± 8.9 *

Each value represents the mean ± S.E. Significantly different from HFD-control: * *p* < 0.05. Sol. M.: Soleus Muscle. ASP: Asperuloside.

**Table 12:** Preliminary test of the preventive effect of forsythia leaf tea against influenza A virus infection.

	Non-Intake Number (%)	Intake Number (%)
Infected	15 (12.2)	6 (7.9)
Non-infected	108 (87.8)	70 (92.1)
Totals	123	76

**Table 13:** Influence of a single oral dose of FLE (6.67 g/kg/day) to male ddYmice on body weight.

Days	0	1	2	3	7	14
Cont (5)	32.9 ± 3.15	29.1 ± 3.59	33.0 ± 3.26	33.4 ± 2.80	35.1 ± 1.88	37.0 ± 1.58
FLE (5)	33.5 ± 3.35	29.1 ± 3.15	34.1 ± 2.97	33.7 ± 3.13	36.1 ± 1.89	37.6 ± 1.67

Each value represents the mean ± S.D. (*n* = 5) (g).

**Table 14:** Influence of repeated oral administration of FLE (0.166 g/kg/day) to male ddY mice on body weight and organ weight.

(n)	BW (g)	Spleen (g)	Liver (g)	Renal (g)	Lung (g)	Heart (g)	Adrenal (mg)	Thymus (mg)	Testis (g)	Brain (g)	Hypophysis (mg)
Cont (5)	37.1 ± 1.00	0.12 ± 0.019	1.8 ± 0.12	0.63 ± 0.023	0.18 ± 0.012	0.17 ± 0.012	5.3 ± 0.87	50.1 ± 2.23	0.23 ± 0.013	0.48 ± 0.025	1.2 ± 0.42
FLE (5)	38.2 ± 1.31	0.12 ± 0.026	1.9 ± 0.13	0.59 ± 0.043	0.19 ± 0.018	0.17 ± 0.008	5.3 ± 0.88	51.9 ± 14.55	0.24 ± 0.027	0.48 ± 0.021	1.3 ± 0.56

Each value represents the mean ± S.D. (*n* = 5).

## Data Availability

The copyright permissions were obtained for uses of Figures and Tables in this review from Japan Society of Medical and Pharmaceutical Sciences for Traditional Medicine.
